# Targeting mitochondria for ovarian aging: new insights into mechanisms and therapeutic potential

**DOI:** 10.3389/fendo.2024.1417007

**Published:** 2024-06-17

**Authors:** Zi-Han Wang, Zhen-Jing Wang, Huai-Chao Liu, Chen-Yu Wang, Yu-Qi Wang, Yang Yue, Chen Zhao, Guoyun Wang, Ji-Peng Wan

**Affiliations:** ^1^ Department of Gynecology, Shandong Provincial Hospital Affiliated to Shandong First Medical University, Jinan, China; ^2^ Jinan Key Laboratory of Diagnosis and Treatment of Major Gynecological Diseases, Shandong Provincial Hospital Affiliated to Shandong First Medical University, Jinan, China; ^3^ Center for Reproductive Medicine, Shandong University, Jinan, China; ^4^ Cancer Center, Renmin Hospital of Wuhan University, Wuhan, China

**Keywords:** mitochondria, ovarian aging, reproductive health, mitochondrial dysfunction, mitochondrial therapies, infertility

## Abstract

Ovarian aging is a complex process characterized by a decline in oocyte quantity and quality, directly impacting fertility and overall well-being. Recent researches have identified mitochondria as pivotal players in the aging of ovaries, influencing various hallmarks and pathways governing this intricate process. In this review, we discuss the multifaceted role of mitochondria in determining ovarian fate, and outline the pivotal mechanisms through which mitochondria contribute to ovarian aging. Specifically, we emphasize the potential of targeting mitochondrial dysfunction through innovative therapeutic approaches, including antioxidants, metabolic improvement, biogenesis promotion, mitophagy enhancement, mitochondrial transfer, and traditional Chinese medicine. These strategies hold promise as effective means to mitigate age-related fertility decline and preserve ovarian health. Drawing insights from advanced researches in the field, this review provides a deeper understanding of the intricate interplay between mitochondrial function and ovarian aging, offering valuable perspectives for the development of novel therapeutic interventions aimed at preserving fertility and enhancing overall reproductive health.

## 1 Introduction

Ovarian aging is a complex process characterized by a gradual decline in both the quantity and quality of oocytes, a phenomenon that has significant implications for female fertility (1). As women age, the impact of aging on the ovaries becomes increasingly pronounced, particularly after the mid-30s (2). This age-related decline in ovarian function not only results in reduced fertility and an increased risk of pregnancy complications but also significantly impacts critical elements like hormonal balance, bone health, cardiovascular well-being, cognitive function (3). Understanding the mechanisms driving ovarian aging is crucial for the development of interventions aimed at preserving fertility and potentially mitigating the impact of age-related fertility decline on women’s overall health.

Mitochondria, widely known as the cellular “powerhouses”, assume a fundamental role in energy generation through oxidative phosphorylation (4). Considering that the oocyte is the body’s most mitochondria-rich cell, these organelles bear significant responsibility in fostering its development, promoting follicular growth, and orchestrating hormone regulation—all crucial factors in ensuring successful reproduction (5). While several other factors, such as vascular network defects, hormonal dysregulation, genetic and epigenetic alterations, and environmental/lifestyle influences, have been identified as contributing to the aging of the ovary, mitochondrial dysfunction appears to be a more central and significant driver of this process (2, 6). Moreover, preserving mitochondrial function not only holds promise for enhancing reproductive outcomes but also for delaying aging in women. Exploring therapeutic strategies targeted at maintaining mitochondrial health could offer significant opportunities for addressing age-related fertility decline and promoting reproductive and overall well-being in women.

In this review, we focus on the intricate interplay between mitochondrial function and ovarian aging, and the mechanisms through which mitochondrial health affects oocyte and ovarian follicle quality, and how it contributes to age-related fertility decline. Furthermore, we explore potential therapeutic strategies aimed at preserving mitochondrial function to enhance reproductive outcomes for women as they age.

## 2 The role of mitochondria in ovarian cell fate

Mitochondria are double-membraned organelles found within the cytoplasm of eukaryotic cells. The outer membrane is porous, allowing the passage of ions and small molecules. In contrast, the inner membrane is impermeable and houses the electron transport chain (ETC), which is a combination of 5 protein-lipid enzyme complexes, electron carriers coenzyme Q10 (CoQ10) and cytochrome c. The ETC generates ATP by transferring electrons to and from these complexes and creating a proton gradient across the inner membrane. Moreover, the inner membrane forms invaginations called cristae, increasing the surface area for energy production (7). The space between the two membranes, known as the intermembrane space, and the innermost compartment of mitochondrion, known as the matrix, are crucial for the transport of protons during oxidative phosphorylation (4). In addition, mitochondria are semiautonomous organelles that have their own DNA. Mitochondrial DNA (mtDNA) contains 37 genes coding for 13 mitochondrial proteins, which are part of the central subunits of the ETC (8).

Cellular senescence, a hallmark of aging, is primarily characterized by an irreversible cell cycle arrest, where upregulation of cyclin-dependent kinase inhibitors such as p16, Rb, p21, and p53 plays a pivotal role (9). This regulatory cessation of cell division is associated with increased expression of genes that enforce this arrest. Concurrently, mitochondrial dysfunction emerges as a critical aspect of aging. Mitochondria are acknowledged not only as the powerhouse of the cell, providing essential energy but also for their involvement in a spectrum of cellular processes including cell cycle regulation, apoptosis, and metabolic control (10, 11). Given the integral nature of these functions, mitochondrial dysfunction undeniably contributes to the aging phenotype. This is particularly relevant in oocytes, which are among the most mitochondria-enriched cells in the organism (5), underscoring the significance of mitochondrial health in ovarian aging and the broader implications for reproductive capacity and overall vitality. Ovarian cells, particularly oocytes and granulosa cells, have high metabolic demands to support their specialized functions, such as oogenesis and steroidogenesis. These cellular processes require mitochondria to provide sufficient energy through oxidative phosphorylation (12, 13). However, ovarian cells possess lower antioxidant defense capabilities compared to other cell types, rendering them more susceptible to mtDNA damage induced by oxidative stress (14). The accumulation of mtDNA mutations and depletion, in turn, exacerbates mitochondrial dysfunction in ovarian cells during the aging process (15).

**Figure 1:**
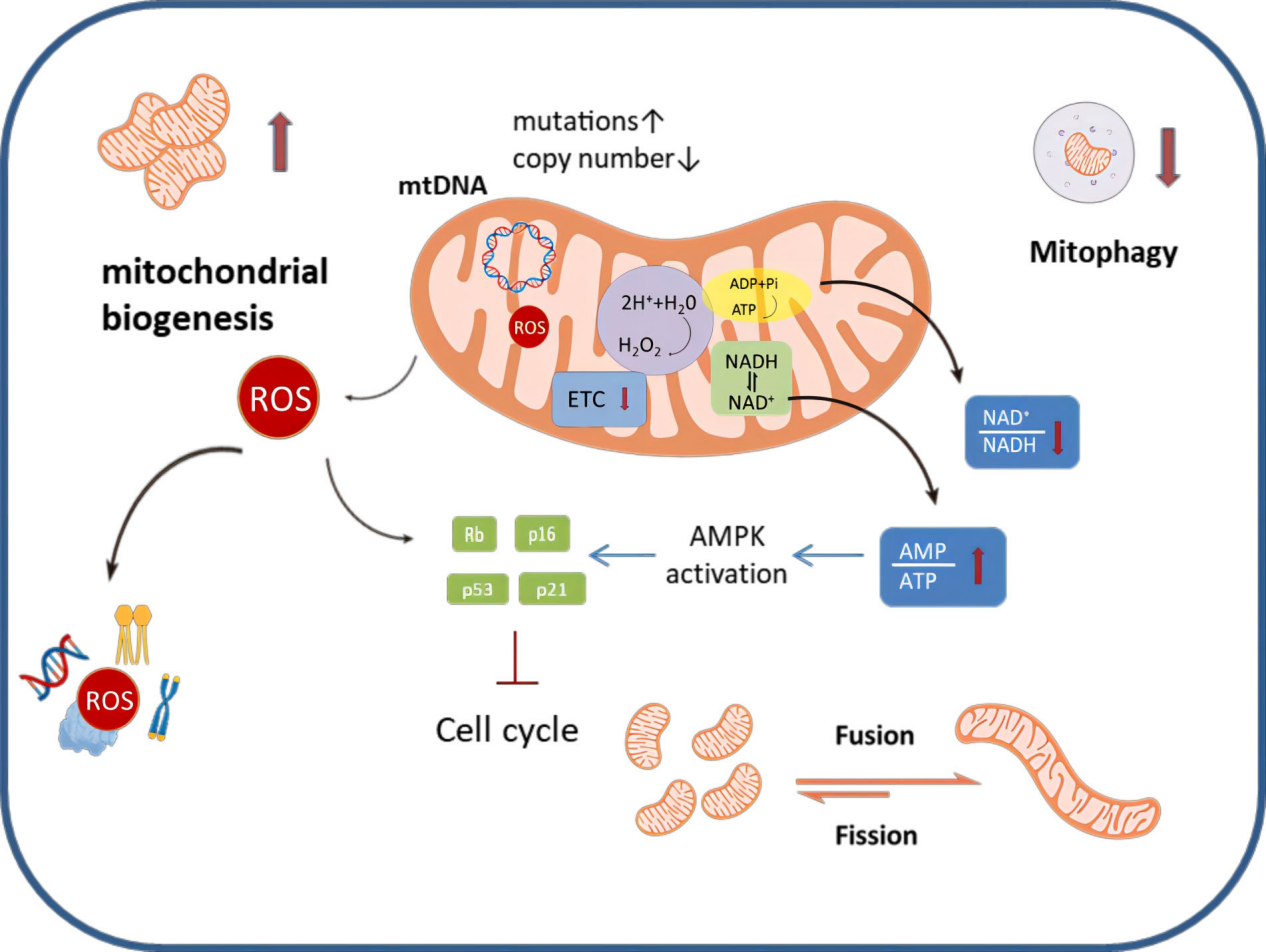
An overview of mitochondrial dysfunction that occurs during ovarian aging. Senescent ovarian cells can be characterized by a number of distinct changes to their homeostatic mechanisms; specifically mitochondrial biogenesis is exacerbated, mitophagy is decreased and mitochondrial dynamics favor fusion, making the mitochondrial networks highly perfused. Metabolically, the ETC is disrupted, this leads to alterations in the AMP/ATP ratio and the NAD+/NADH ratio, as well as enhanced production of ROS.ROS can damage mitochondria as well as induce nuclear DNA damage. Similarly, ROS can cause damage to proteins, lipids and other components, affecting the normal function of ovarian cells.MtDNA mutation increased and mtDNA copy number decreased in senescent ovarian cells.

Nearly two decades ago, Hayflick et al. elucidated the connection between mitochondria and the cellular senescent phenotype (16). A growing body of evidence supports the notion that senescent ovarian cells undergo structural, functional and dynamic changes in their mitochondria (17) (**Figure 1**). For example, senescent cumulus cells demonstrate increased abnormal mitochondrial numbers and decreased mitochondrial activity, characterized by reduced mitochondrial membrane potential, elevated proton leakage, and heightened generation of reactive oxygen species (ROS) (18, 19). Furthermore, senescent ovarian cells also show dramatic changes in mitochondrial morphology. The mitochondria in these cells are in a state of hyper-fusion, while a healthy mitochondrial network constantly undergoes fission and fusion events to meet metabolic needs and allow for the removal of dysfunctional entities (20).

The following sections will elucidate the detailed mechanisms by which mitochondrial dysfunction influences the senescence phenotype in ovarian cells.

### 2.1 Mitochondria and ROS balance

Free Radical Theory of Aging, introduced in 1956, proposes that the accumulated damage caused by free radicals, which are produced during normal metabolic processes, contributes to the aging process (21). ROS, including free radicals, are believed to induce oxidative damage, affecting cellular structures and molecules over time (22). These ROS also act as signaling molecules, influencing various cellular processes such as cell differentiation, proliferation, and apoptosis. However, an elevation in ROS production, often linked to mitochondrial dysfunction, can result in oxidative stress, leading to harm to cellular components and contributing to the aging processes (23).

**Figure 2:**
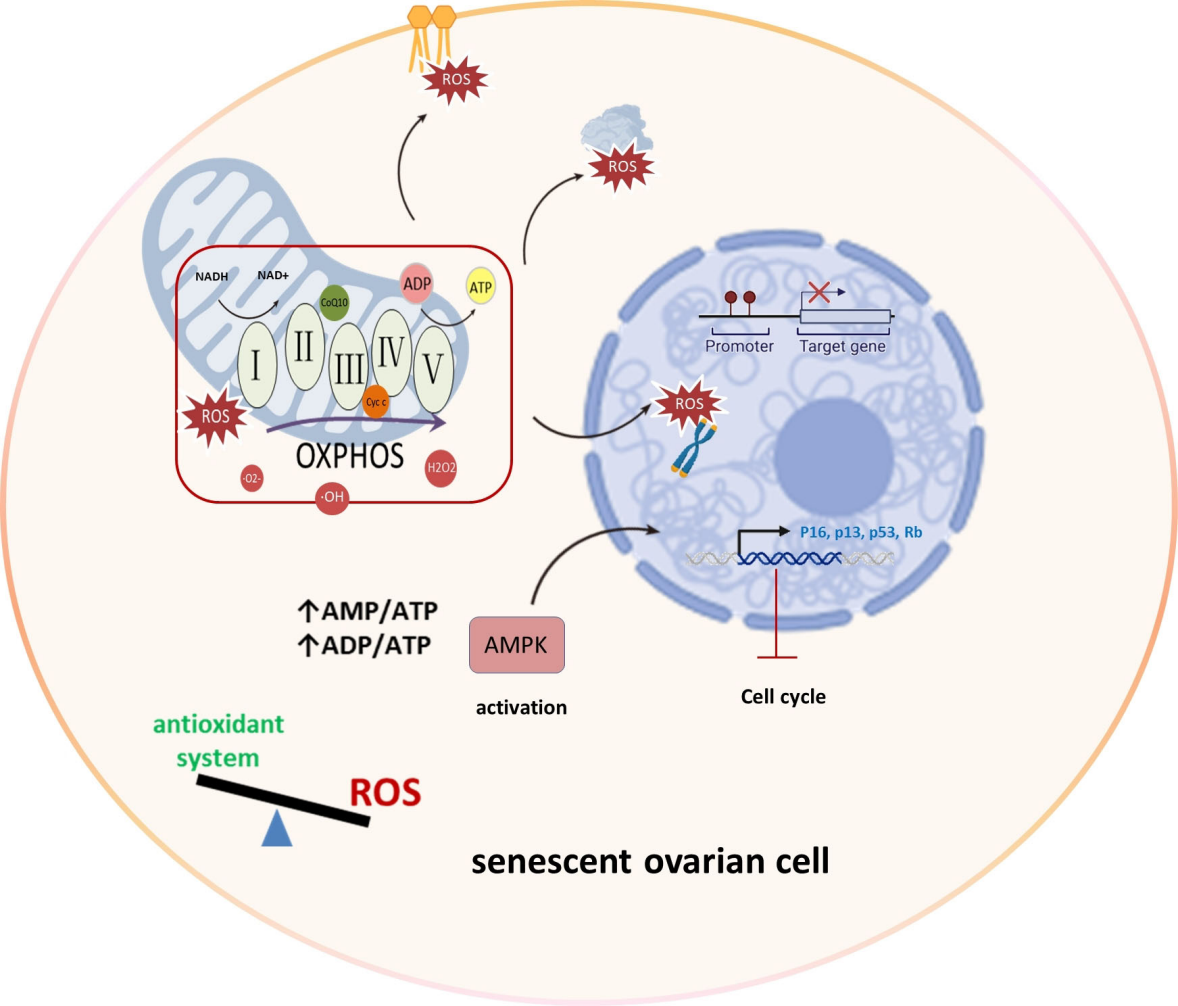
Mitochondrial OXPHOS and ROS production in ovarian aging. In the process of ovarian aging, the balance between oxidation and antioxidant systems is disrupted. Mitochondria display aberrant OXPHOS that produces excessive amounts of ROS that damage lipids, proteins and DNA, specially telomeric regions. And reduced ATP level activates AMPK leading to cell cycle arrest and senescence induction. Oxidative phosphorylation (OXPHOS), AMP-activated protein kinase (AMPK), Reactive Oxygen Species (ROS), superoxide (·O2-), hydrogen peroxide (H2O2), and hydroxyl radicals (·OH), Retinoblastoma protein (Rb).

Mitochondria primarily produce superoxide, hydrogen peroxide, and hydroxyl radicals through the electron transport chain during oxidative phosphorylation, serving as major ROS sources (24). The mechanism underlying ROS-induced aging involves the development of a growth arrest phenotype due to mitochondrial damage, excessive ROS generation, and activation of the DNA damage response through the p53/p21 pathway (25). This imbalance between ROS production and antioxidant systems can lead to oxidative stress, causing damage to lipids, proteins, and DNA, particularly in telomeric regions. Moreover, this imbalance hampers ATP production, triggering AMP-activated protein kinase (AMPK) activation, which in turn leads to cell cycle arrest and the induction of senescence (26). Elevated ROS levels, particularly in the ovaries, can impact cellular components, leading to diminished ovarian reserves and affecting oocyte developmental competence (27). Understanding these intricate connections between mitochondria and ROS is crucial for unraveling the complexities of ovarian aging and reproductive longevity (**Figure 2**).

The study by Lim highlights the correlation between aging and increased oxidative damage in the ovarian tissue, coupled with a decline in antioxidant gene expression (28). Additionally, research by Smits et al. confirms oxidative damage and mitochondrial dysfunction in oocytes of advanced maternal age, even at the primordial follicle stage (29).

Sirtuins, being a family of nicotinamide adenine dinucleotide (NAD+)-dependent histone deacetylases, consist of seven SIR2 homologs (SIRT1-7) in mammals (30). Sirt3 is specifically located in the mitochondrial matrix and plays a crucial role in enhancing mitochondrial resilience to ROS (31). Deficiency of Sirt3 in female mice accelerates ovarian aging, affecting mitochondrial function and oocyte quality (32). Within mitochondria, peroxiredoxin 3 (Prdx3) regulates mitochondrial ROS (33), and reduced Prdx3 expression in aged mouse oocytes increases susceptibility to oxidative stress (27).

Granulosa cells (GCs), constituting the predominant cell population in the ovary, require stable mitochondria for their growth, proliferation, and division (34). Tamura et al. reported a notable decrease in the proportion of active mitochondria in an oxidative stress-induced model of GCs (35). Mitochondrial dysfunction disrupts the bi-directional interaction between oocytes and GCs, hindering cell growth and development (5). Additionally, mutations in Immp2l, which encodes a protein integral to the inner mitochondrial membrane, contribute to ovarian aging via the ROS-Wnt/β-catenin pathway, exacerbating oxidative stress in GCs (36).

### 2.2 Mitochondrial quality control

Mitochondrial quality control is a fundamental cellular process essential for preserving the integrity and functionality of mitochondria. This regulatory network encompasses three key mechanisms: mitochondrial biogenesis, fusion and fission dynamics, and mitophagy.

Mitochondrial biogenesis involves the growth of functional mitochondria by producing new components like proteins, lipids, and mtDNA. These processes are crucial for cellular energy production, adapting to changes in energy demands, and the overall health of cells and tissues. Mitochondria have their own DNA (mtDNA) and hence biogenesis of mitochondria requires a coordination of nuclear DNA and mtDNA, both of which encode for mitochondria proteins (37). Notably, mitochondrial biogenesis plays a critical role in supporting the energy requirements of growing oocytes and determining the size of the follicular pool in the ovaries, which is a key factor in ovarian aging and reproductive longevity (38).

The dynamic balance of mitochondrial dynamics involving fission and fusion is essential for maintaining mitochondrial function (39). The fusion of damaged and undamaged mitochondria can act as a defensive mechanism, diluting the extent of damage. Conversely, mitochondrial fission facilitates the isolation and subsequent elimination of impaired mitochondria through mitophagy (40). Therefore, the meticulous orchestration of fission and fusion is of paramount importance for the health of oocytes and follicles (41). Research findings have demonstrated that the loss of the mitochondrial fission protein Dynamin-related protein 1 (Drp1), which is encoded by the gene Fission 1 (Fis1), results in a decline in oocyte quality (42). Mitofusins, particularly Mitofusin 1 (Mfn1) and Mitofusin 2 (Mfn2), are integral to the process of mitochondrial fusion and play essential roles in ovarian function and female fertility. Oocyte-specific deletion of Mfn1 has been associated with diminished ovarian follicular reserve, emphasizing its crucial role in maintaining ovarian function and fertility (43). Additionally, Mfn2 has been linked to the regulation of oocyte development and quality through its influence on meiosis and mitochondrial function (44).

Mitophagy, the process of degrading damaged or dysfunctional mitochondria, plays a crucial role in maintaining cellular health by regulating mitochondrial numbers and ensuring a population of healthy mitochondria (41). Successful mitophagy necessitates mitochondrial fission, which segregates the compromised mitochondria. These are then encased in autophagosomes and subsequently merged with lysosomes for degradation (45). Defective mitophagy can lead to the accumulation of damaged mitochondria, contributing to increased oxidative stress and impaired energy production in the ovaries, consequently affecting oocyte quality and fertility.

**Figure 3:**
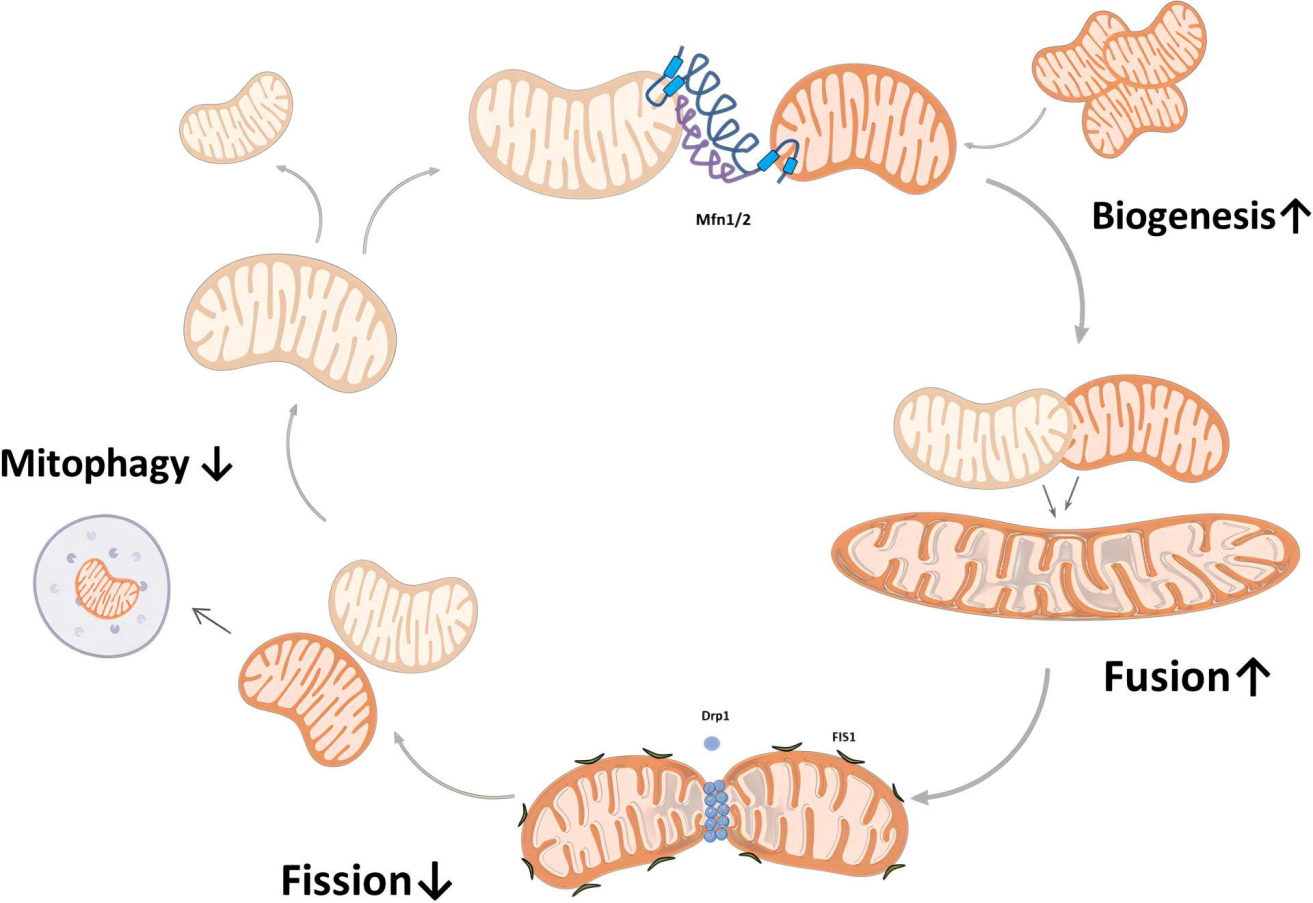
Mitochondrial quality control in ovarian aging. During ovarian aging, there is a discernible deterioration in mitochondrial quality control, characterized by enhanced mitochondrial biogenesis, a shift in dynamics favoring fusion over fission, and a decrease in mitophagy. Asa a consequence, senescent ovarian cells display an increased mitochondrial mass and mitochondria with a hyperfused and elongated morphology. Mitofusin 1 (Mfn1), Mitofusin 2 (Mfn2), Dynamin-related protein 1 (Drp1).

During aging, there is a discernible deterioration in mitochondrial quality control, characterized by enhanced mitochondrial biogenesis, a shift in dynamics favoring fusion over fission, and a decrease in mitophagy (46). This progressive decline leads to an accumulation of mitochondria within senescent ovarian cells, reflected by an increased mitochondrial mass, a heightened state of fused mitochondria, and an elongated mitochondrial morphology (47) (**Figure 3**).

Overall, mitochondrial quality control holds significant importance in preserving ovarian health and managing fertility issues associated with aging. Understanding and maintaining the delicate balance of mitochondrial biogenesis, dynamics, and mitophagy is critical for addressing ovarian aging and preserving fertility (37, 48, 49).

### 2.3 Mitochondrial DNA dysfunction and ovarian aging

Mitochondrial DNA (mtDNA) is a unique genetic material inherited exclusively from the mother, encoding 13 core proteins critical for the efficiency of the mitochondrial energy-generating system (50). The dysfunction of mtDNA, whether quantitative or qualitative, may arise from various factors such as oxidative stress, environmental factors, and the natural aging process (51). Additionally, due to its proximity to the ETC and the consequent generation of ROS, mtDNA may be more susceptible to mutational burden, with a mutation rate approximately 25 times higher than nuclear DNA (52).

Ross et al. explore the association of acquired and inherited mtDNA mutations with premature aging, particularly focusing on ovarian aging, using a series of mouse mutant models. Their findings reveal that maternally transmitted mtDNA mutations induce mild aging phenotypes in mice with a normal nuclear genome, anticipating reduced fertility and exacerbating premature aging phenotypes (53). Additionally, acquired (somatic) mtDNA mutations in humans may result from oxidative damage or the inherent error rate of mtDNA polymerase gamma (54, 55). Human oocytes have been found to harbor mitochondrial point mutations, with a higher accumulation in aged oocytes, as indicated by the analysis of human oocytes for specific mutations (50).

Animal studies have shown that lower mtDNA copy number is associated with aging, as evidenced by old mice (56) and cows (57) with decreased fecundity having significantly less mtDNA in their oocytes compared to younger counterparts. Furthermore, the association between mtDNA copy number in cumulus cells and reproductive potential has been observed in human studies, indicating its relevance to the reproductive potential of women of advanced reproductive age (58). XY Zhou et al. reported that elevated levels of cell free mtDNA may be associated with premature ovarian insufficiency, shedding light on the potential role of mitochondrial dysfunction in the pathophysiology of this condition (59).

### 2.4 Mitochondrial unfolded protein response

The mitochondrial unfolded protein response (UPRmt) is a cellular mechanism designed to maintain mitochondrial homeostasis by addressing the accumulation of unfolded or misfolded proteins within the mitochondrial matrix (60). Activated in response to mitochondrial stress, UPRmt orchestrates processes such as protein refolding, degradation of damaged proteins, and the promotion of mitochondrial biogenesis to ensure a stable mitochondrial environment (61). ClpP (caseinolytic peptidase P), a protease enzyme located in the mitochondrial matrix, plays a key role in UPRmt (62).

Studies have shown that ClpP deficiency can lead to impaired mitochondrial stress response, increasing apoptosis in GCs, and negatively impacting ovarian function and reproductive health (63, 64). Moreover, the absence of ClpP also accelerates the depletion of ovarian follicle reserve by over-activating the mTOR pathway (65). Under electron microscopy, mitochondria in Clpp-deficient cumulus cells have a smaller aspect ratio (length/width) and have a larger coverage area (mitochondrial area/cytoplasmic area). These ultrastructural changes were accompanied with diminished expression of mitochondrial dynamics genes (66). This underlines the intricate relationship among ClpP, mitochondrial dynamics, and reproductive health.

## 3 Therapeutic approaches

Targeting mitochondrial dysfunction represents a promising tool to prevent ovarian aging. Here, we explore the potential of targeting mitochondrial dysfunction through innovative therapeutic approaches, including antioxidants, metabolic improvement, biogenesis promotion, enhanced mitophagy, mitochondrial replacement therapy (MRT) and traditional Chinese medicine (TCM) treatments. Also, growth hormone appears to enhance mitochondrial function and reduce aneuploidy in aged mice, highlighting its potential as a therapeutic avenue for mitigating ovarian aging and enhancing reproductive outcomes (67–69). We summarize the current major mitochondrial therapies in **Table 1**, and while they show great potential in alleviating ovarian aging, there are still some challenges and limitations in practical application.

**Table 1:** Mito-therapies to prevent ovarian aging.

Classification	Representative	Mechanism	Ref.
Decreasing mitochondrial ROS level	resveratrol	activate pathways such as Nrf2-ARE	(70)
	melatonin	enhance antioxidant defense	(71)
	SDG	notable scavenging ability against ROS	(72)
	MitoQ	antioxidant specifically directed to mitochondria	(73)
	α-KG	reduce ROS accumulation	(74)
Improving mitochondrial metabolism	NMN NAM NR	raise NAD+ levels and promote TCA cycle	(75)
Enhancing mitochondrial biogenesis	resveratrol	activate the AMPK/SIRT1/PGC-1α axis	(76)
	HBP1	regulate gene expression, protein interactions, and cellular physiological processes involved in mitochondrial biogenesis.	(77)
Promoting efficient mitophagy	UA	activate mitophagy through the PINK1-Parkin Pathway	(78)
	metformin	upregulate of essential mitophagy-related genes	(79)
Mitochondrial replacement therapy	AMT	extract healthy mitochondria from the patient’s own cells and transplant them into their dysfunctional cells	(80)
	CT	the injection of cytoplasm, containing healthy mitochondria, from a donor’s cell into a recipient’s cell	(81)
	GNT	transfer the genetic material of a patient’s oocyte/zygote with compromised cytoplasm to the cytoplasm of an enucleated oocyte/zygote of a healthy donor	(82)
Traditional Chinese medicine	QZD	enhance ovarian mitochondrial function and regulate ovarian mitochondrial apoptosis	(83)
	Astragalus Angelica Panax ginseng	increase antioxidant capacity and reduce apoptosis	(84)

SDG, secoisolariciresinol diglucoside; MitoQ, Mitochondrially-targeted coenzyme Q; α-KG, alpha-ketoglutarate; NMN, nicotinamide mononucleotide; NAM, nicotinamide; NR, nicotinamide riboside; HBP1, HMG-box transcription factor 1; UA, Urolithin A; AMT, Autologous mitochondrial transplantation; CT, Cytoplasmic transfer; GNT, Germline nuclear transfer; QZD, Qingxin Zishen decoction.

### 3.1 Decreasing mitochondrial ROS level

Mitochondrial-targeted antioxidant therapies have garnered significant attention as a key strategy to alleviate the adverse impact of mitochondrial ROS accumulation, given their central role in driving ovarian aging (85, 86). The antioxidant system comprises a complex interplay of various components, including both enzymes and non-enzymatic substances. Key enzymes within this system encompass superoxide dismutase (SOD), catalase (CAT), glutathione (GSH) and glutathione peroxidase (GSH-Px). Non-enzymatic elements predominantly consist of melatonin, as well as vitamins C, D, and E, and essential trace elements such as copper, zinc, and selenium (27). Consequently, interventions involving the supplementation of antioxidants like vitamins C, D, and E, upregulation of SOD and CAT through sirtuins (87), and the administration of chemical and natural compounds with documented antioxidant properties (e.g., phycocyanin (88), fisetin, and curcumin) have been employed to mitigate oxidative stress and inhibit cellular senescence across various cell types.

Focusing on ovarian cells, interventions involving resveratrol, melatonin, and Secoisolariciresinol diglucoside (SDG) have shown efficacy in reducing intracellular ROS levels. Resveratrol, a compound found in plants like grapes and berries, has gained attention for its potential health benefits, particularly its anti-aging properties (89). Studies suggest that resveratrol can modulate oxidative stress mechanisms, potentially slowing follicular atresia and mitigating the effects of aging on ovarian tissues (90, 91). Its purported effects involve the activation of pathways such as Nrf2-ARE, thereby enhancing antioxidant defense and reducing oxidative damage (70). Similarly, melatonin has emerged as a pivotal player in addressing ovarian aging (92). Studies indicate its potential in ameliorating age-related ovarian decline by enhancing antioxidant defense, protecting oocytes from damage caused by oxidative stress, and preserving mitochondrial function, ultimately impacting ovarian tissue health, follicular development, and oocyte quality (71). Additionally, SDG has exhibited notable scavenging ability against ROS, which gradually accumulates in ovarian tissues. Research by He et al. in 2021 suggests that SDG possesses beneficial effects on aging ovaries, as it has been shown to improve ovarian reserve by inhibiting oxidative stress (72).

Furthermore, mitochondrial-targeted coenzyme Q10 (MitoQ), a unique antioxidant specifically directed to mitochondria, has demonstrated protective effects against oxidative damage in ovarian tissue. ​Such protection suggests a potential to retard the process of ovarian aging, highlighting MitoQ’s significance in reproductive health interventions (73). A recent study conducted by Wang et al., showed that the supplementation of alpha-ketoglutarate (α-KG) to aging mice over a 4-month period demonstrated marked improvements in ovarian reserve, manifested by increased follicle numbers and enhanced oocyte quality. These benefits were evidenced by reduced fragmentation rates, decreased ROS levels, and a reduced frequency of abnormal spindle assembly (74). Furthermore, α-KG administration exhibited positive effects on post-ovulated aging oocyte quality and early embryonic development by enhancing mitochondrial functions, and reducing ROS accumulation and abnormal spindle assembly.

### 3.2 Improving mitochondrial metabolism and enhancing mitochondrial biogenesis

Recent researches have elucidated that during cell aging, metabolism shifts towards increased mitochondrial oxidative metabolism, accompanied by enhanced mitochondrial biogenesis (93, 94). This metabolic shift results in a decline in NAD+ levels and an elevation in AMP and ADP. Although the increase in mitochondrial biogenesis can offset the loss of healthy mitochondria due to mitochondrial stress, sustained stress experienced by mitochondria during cellular senescence may expose newly synthesized mitochondria to oxidative stress, creating a positive feedback loop that enhances ROS production and exacerbates mitochondrial damage. Therefore, adopting strategies to improve mitochondrial metabolism, enhance mitochondrial biogenesis, and limit ROS production is critical in preventing ovarian cell senescence and prolonging reproductive life (95).

Low levels of NAD+ have been demonstrated to be associated with mitochondrial metabolism disorders and the senescence of stem cells, whereas elevated NAD+ levels have been linked to enhanced mitochondrial function (96, 97). The study by Yang et al. found that the deletion of enzymes responsible for *de novo* NAD (+) biosynthesis led to a significant acceleration of ovarian aging, highlighting the pivotal role of these enzymes in maintaining ovarian health and function (98). Correspondingly, compounds that raise NAD+ levels, such as nicotinamide mononucleotide (NMN), nicotinamide (NAM), and nicotinamide riboside (NR), acting as precursors of NAD+, are acknowledged for their potential in retarding the aging process (75).

Several interventions have also shown promise in stimulating mitochondrial biogenesis and combatting ovarian aging, including exercise (99), controlled exposure to ROS, and nutritional therapy (100), involving specific nutrients such as CoQ10 (101) and resveratrol. In addition to its well-known antioxidant properties, resveratrol also plays a crucial role in enhancing mitochondrial biogenesis by activating the AMPK/SIRT1/PGC-1α axis, with PGC-1α being the key regulatory factor of mitochondrial biogenesis (76, 102). These approaches may offer potential avenues for maintaining ovarian health and counteracting age-related ovarian decline.

In addition, researches have shown that the tumor suppressor High Mobility Group (HMG)-box transcription factor 1 (HBP1) enhances mitochondrial function, reduces apoptosis of GCs, and significantly preserves ovarian reserve by regulating gene expression, protein interactions, and cellular physiological processes involved in mitochondrial biogenesis. These findings underscore the consequential role of HBP1 in extending the reproductive lifespan (77).

While increased mitochondrial biogenesis can be a beneficial adaptive response, uncontrolled or excessive biogenesis can also lead to deleterious consequences, such as increased NADH, decreased NAD+, and elevated AMP and ADP levels (103). Therefore, a careful balance must be struck when targeting mitochondrial biogenesis as a strategy to improve mitochondrial metabolism and function in the context of ovarian aging.

### 3.3 Promoting efficient mitophagy

Enhancing mitophagy is an area of active research with potential therapeutic implications for addressing age-related fertility issues and improving reproductive health (104–106). Therapeutic interventions aimed at enhancing mitophagy encompass lifestyle modifications, dietary therapies, and pharmacological approaches.

Physical activity has been associated with a greater expression of mitochondrial fission and mitophagy markers, indicating its positive impact on mitochondrial quality control systems (107). Conversely, overfeeding is linked to the accumulation of impaired mitochondria and oxidative stress, underscoring the influence of lifestyle on mitophagy and mitochondrial health (108).

A variety of natural dietary compounds have been identified as potential agents in promoting mitophagy. Polyphenols, flavonoids, spermidine, and trehalose, along with compounds like resveratrol and Urolithin A(UA), are known to facilitate the restoration of normal mitophagy fluxes (109). Resveratrol effectively reinstates mitochondrial autophagy, contributing to the health of mitochondria in ovarian cells (110). UA, in particular, activates mitophagy through the PINK1-Parkin Pathway, which plays a crucial role in enhancing oocyte quality and extending reproductive functionality (78). Recently, Bhansali et al. found that metformin facilitates mitophagy through upregulation of essential mitophagy-related genes, thereby normalizing mitochondrial function (79). This positions metformin as a contender in the effort to decelerate ovarian aging and sustain reproductive health.

There is a complex relationship between mitochondrial quality control and reproductive lifespan. For instance, the daf-2 mutant model has been highlighted for its ability to sustain punctate mitochondrial morphology and execute fission alongside PINK1-mediated mitophagy, processes essential for maintaining oocyte integrity over time (111). Moreover, research has established a connection between elevated PGAM5 expression and age-related changes in mitochondrial dynamics in ovarian cells, suggesting a possible role for PGAM5 in maintaining and regulating mitochondrial function as organisms age (112). However, the development of therapeutic strategies that delay ovarian aging by manipulating mitochondrial dynamics remains an area in need of further exploration.

### 3.4 Mitochondrial replacement therapy

Mitochondrial replacement therapy (MRT) has emerged as an investigative technique to address mitochondrial dysfunction and its role in ovarian aging and poor oocyte quality. The premise of MRT is to replace defective mitochondria within an oocyte with healthy mitochondria, thereby improving the bioenergetic capacity and overall quality of the oocyte (113).

The primary methods of MRT include autologous mitochondrial transplantation (AMT), cytoplasmic transfer(CT), and germline nuclear transfer(GNT) (114). AMT involves extracting healthy mitochondria from the patient’s own cells and transplanting them into their dysfunctional cells, with the aim of restoring mitochondrial function and improving cellular bioenergetics (80). CT entails the injection of cytoplasm, containing healthy mitochondria, from a donor’s cell into a recipient’s cell (81). GNT offers the possibility to transfer the genetic material of a patient’s oocyte/zygote with compromised cytoplasm to the cytoplasm of an enucleated oocyte/zygote of a healthy donor (82).

In addition to these established techniques, other experimental approaches to MRT are being explored. The CRISPR/Cas9 gene editing technology holds potential for repairing mutated mtDNA in women with aging ovaries (115). Another proposed approach involves the use of stem cells to produce “young” mitochondria. The theory behind this method is that stem cells could be leveraged to generate a fresh supply of healthy mitochondria, which could then be used to supplement or replace dysfunctional mitochondria in aging oocytes (116). However, rigorous testing and evaluation will be essential to ensure the responsible development and implementation of these innovative therapies.

These mitochondrial replacement technologies offer potential avenues for addressing mitochondrial-related disorders and age-related fertility decline, but they also raise important social and ethical considerations (117). Critics argue that MRT, in particular, raises ethical concerns, as it involves the creation of embryos with genetic material from three individuals-the nuclear DNA from the intended parents and the mtDNA from the donor (118). There are also concerns about the long-term safety and efficacy of these interventions, the potential for genetic modifications, and the commercialization and equitable access to these treatments. Careful deliberation and oversight by relevant regulatory bodies and bioethical committees are necessary to address these complex issues (119).

### 3.5 Traditional Chinese medicine

Traditional Chinese medicine (TCM) has long been recognized for its potential in addressing various health concerns, including the issue of ovarian aging. TCM offers a unique perspective and therapeutic approaches to regulate ovarian aging, with a particular emphasis on the role of mitochondria. Some of the key mechanisms through which TCM may contribute to the regulation of ovarian aging include enhancing mitochondrial biogenesis, improving mitochondrial function, modulating mitochondrial dynamics, and regulating mitochondrial quality control (120). For instance, the Qingxin Zishen decoction (QZD) has shown promise in delaying ovarian aging and mitigating age-related ovarian conditions by enhancing ovarian mitochondrial function and regulating ovarian mitochondrial apoptosis in older rats (83), providing potential treatment options for age-related ovarian changes.

Numerous studies have explored the potential of TCM in addressing ovarian aging and related reproductive health concerns. Herbal formulations, such as those containing ingredients like Astragalus, Angelica, and Panax ginseng, have shown promising results in improving ovarian function, increasing the number of follicles, and enhancing oocyte quality by elevating antioxidant capacity, reducing apoptosis and other mechanisms in both animal models and human clinical trials (84, 121). Furthermore, TCM-based interventions have been investigated for their ability to alleviate the symptoms associated with ovarian aging, such as menopausal symptoms, infertility, and the risk of age-related gynecological conditions (122).

While the primary focus of TCM in addressing ovarian aging is on regulating the function and health of the ovaries, the holistic nature of TCM suggests that its interventions may have a broader impact on the body as a whole. TCM practitioners believe in the interconnectedness of all organs and systems within the body, with the goal of restoring balance and harmony. By targeting the underlying imbalances that contribute to ovarian aging, TCM therapies, such as herbal remedies, acupuncture, and lifestyle modifications, may have a positive influence on the overall health and functioning of other organs as well (123).

### 3.6 Therapeutic challenges in targeting ovarian mitochondria

The mitochondria have emerged as a promising target for regulating ovarian aging, but there are several challenges and limitations that need to be considered. One key challenge is the significant heterogeneity observed in ovarian cells, with respect to their mitochondrial content, morphology, and function (124). This diversity within the ovarian tissue makes it difficult to develop a “one-size-fits-all” approach to mitochondrial-targeted therapies. Another challenge is the effective delivery of mitochondrial-targeted compounds, including some TCM herbs and formulations, to the mitochondria of ovarian cells (125). Developing appropriate delivery systems and compounds is crucial for maximizing the therapeutic potential.

Assessing the impact of mitochondrial-targeted interventions on ovarian function and aging is also difficult, as it requires sophisticated techniques to accurately measure mitochondrial parameters, such as quality control mechanisms including biogenesis and dynamics, within the ovarian tissue. Furthermore, ovarian aging is a multifactorial process, and individual variations in genetic, environmental, and lifestyle factors may influence the mitochondrial function and the responsiveness to certain interventions. Personalized approaches may be necessary to optimize the therapeutic outcomes (126).

Many promising mitochondrial-targeted therapies have also been limited by their potential for off-target toxicity, as mitochondria are essential for the normal functioning of all cells in the body (127). A deeper understanding of the unique metabolic and functional properties of ovarian mitochondria is needed to develop highly specific mitochondrial-targeted therapies that can selectively accumulate in ovarian cells and avoid off-target effects in other tissues.

In summary, considering the heterogeneity of ovarian mitochondria, effective delivery concerns, the need for personalized approaches, and off-target toxicity, a comprehensive and multifaceted approach may be necessary to overcome these obstacles and unlock the full potential of mitochondrial-targeted therapies for addressing ovarian aging.

## 4 Conclusion

Mitochondria play a central role in ovarian aging and reproductive health. Elevated reactive oxygen species, impaired mitochondrial quality control network, dysfunctional mtDNA, and decreased efficiency of mitochondrial unfolded protein response collectively contribute to ovarian cell aging. Therapeutic interventions targeting mitochondrial dysfunction are critical to protect ovarian function and support overall reproductive health. In the future, there should be a deeper understanding of the role of mitochondria in ovarian aging and further optimization of treatment methods that target mitochondria to fully utilize the potential of mitochondrial therapies.

## Author contributions

Z-HW: Writing – original draft, Writing – review & editing. Z-JW: Writing – review & editing. H-CL: Writing – review & editing. C-YW: Writing – review & editing. Y-QW: Writing – review & editing. YY: Writing – review & editing. CZ: Writing – review & editing. G-YW: Writing – review & editing. J-PW: Writing – original draft, Writing – review & editing.
